# Subacute Hepatotoxicity of Extracts of *Senna occidentalis* Seeds in Swiss Albino Mice

**DOI:** 10.1155/2020/8843044

**Published:** 2020-08-27

**Authors:** Egziharia Mokonen Gebrezgi, Mebrahtom Gebrelibanos Hiben, Kidanemariam Gaim Kidanu, Amanuel Tesfay Tsegay

**Affiliations:** ^1^Department of Anatomy, College of Health Sciences, Aksum University, Axum, Ethiopia; ^2^Department of Anatomy, College of Health Sciences, Mekelle University, Mekelle, Ethiopia; ^3^Department of Pharmacognosy, College of Health Sciences, Mekelle University, Mekelle, Ethiopia

## Abstract

*Senna occidentalis* is potentially toxic to humans and animals. Its seeds are crop contaminant weeds in some localities where liver disease is prevalent. This study assessed the subacute hepatotoxicity of *S. occidentalis* seeds in mice model. Three groups of female Swiss Albino mice (25–28 g, aged 8–10 weeks) received distilled water (control), 400, and 1000 mg/kg extract of *S. occidentalis* seed, respectively. At the end of the study, body weight and liver organ weight were recorded, and tissue and blood samples were collected and analyzed. The results indicated that the extract treated groups, at both doses, showed significant (*p* ≤ 0.001) decrease in mean body weight gain in the fourth week of the experiment. Besides, the extract treated groups showed significant (*p* ≤ 0.001) elevation of liver enzyme markers: alanine aminotransferase and aspartate aminotransferase. Also, histopathological examinations of liver tissue showed moderate microvesicular steatosis of hepatocytes and mild inflammation in the 400 mg/kg treated group as well as marked micro- and macrovesicular steatosis, focal area necrosis, and periportal inflammation with mononuclear cell infiltration in the 1000 mg/kg treated group. Thus, these findings show that *S. occidentalis* seeds exhibit hepatotoxicity in mice, characterized by changes in liver tissue architecture and liver enzyme levels.

## 1. Introduction

The genus *Senna* belongs to the family Fabaceae (Leguminosae). It is one of the largest genera comprising 300–350 species [1]. *Senna* species are commonly used as shade plants; ornamentals, famine foods, and many species are used in both traditional and modern medicines [2]. Despite a wide range of potential medicinal importance of many herbal products, exposure to some of such botanicals may pose a risk of toxicity to both humans and animals [3, 4]. Likewise, some *Senna* species, mainly their seeds, are reported to cause various forms of toxicities regardless of their numerous potential medicinal values [1]. *Senna occidentalis* (Syn: *Cassia occidentalis*) is a small pan-tropical shrub, commonly known as coffee *Senna* [1, 5]. In some poor countries, the dried mature beans/seeds of *S. occidentalis* are roasted and used as coffee substitute [6]. It is native to the tropical regions of America and naturalized in Australia, East Africa, and southern and eastern USA [4, 7, 8]. Various parts of *S. occidentalis* (seeds, roots, leaves, and stems) are traditionally claimed to be useful in treating different infections and other aliments [9–14]. Moreover, some toxicity studies indicate the possible safe use of *S. occidentalis* different plant parts such as the leaves and stems [10]. However, despite many medicinal claims, mainly *S. occidentalis* seeds have been reported to be poisonous to many animal species and humans [1].

Ingestion of large amounts of *S. occidentalis* seeds by grazing animals was reported to cause serious illness and death. Gross lesions in the poisoned animals consisted of necrosis of skeletal muscle fibres, hepatic centrilobular necrosis, and, less frequently, renal tubular necrosis [15]. Likewise, *S. occidentalis* seeds caused several outbreaks in Brazil resulting in lethal poisoning of cattle. The main gross changes in the poisoned cattle were degenerative myopathy in skeletal muscles of the hind limbs and the histology of the affected cattle showed myocardial, hepatic, and renal lesions, and occasionally splenic lesions [16]. Moreover, sudden death of horses due to ingestion of *S. occidentalis* seeds was associated with clinical signs of hepatoencephalopathy and frequent deaths followed by hepatocellular pericentrolobular necrosis and cerebral edema [17]. Induction of mitochondrial damage has been suggested as the key mechanism by which the seeds produce toxic effects on different organs such as the liver of different animal species [1]. Also, although there is little report on human toxicity, clinical spectrum and histopathology of *S. occidentalis* poisoning in children of Western Uttar Pradesh of India were similar to those of animal toxicity, affecting mainly hepatic and skeletal muscle and brain tissues [18]. Different phytochemical groups have been identified in *S. occidentalis* seeds including alkaloids, carbohydrates, flavonoids, glycosides, saponins, tannins, phenols, phytosterols, proteins, amino acids, oils, and fats, as well as other miscellaneous constituents such as N-methylmorpholine. The specific toxin responsible for *S. occidentalis* seed caused toxicity has not been clearly identified. Nevertheless, N-methylmorpholine, toxic alkaloids, anthraquinone derivatives, and toxalbumins were reported as possible candidates [1]. Thus, considering that *S. occidentalis* is a common contaminant weed of agricultural products [1] and that it is a potential crop contaminant weed in northwestern Tigray of Ethiopia where liver disease is prevalent, the present study aimed to investigate the hepatotoxicity effect of the seeds of this plant using mice model since scientific studies are lacking that verify its potential hazard and risk of hepatotoxicity in these localities.

## 2. Methodology

### 2.1. Plant Material Collection and Preparation of Extracts

*S. occidentalis* seeds were collected as per standard techniques of plant collection [19] from Northwestern Tigray of Ethiopia. The plant was authenticated at Mekelle University Department of Biology where a voucher specimen (EM001/2017) was deposited. The seeds were taken out of the dried pods and subsequently powdered using coffee grinder. 500 g of powdered seeds was macerated for 72 hrs using 80% methanol with occasional shaking. The extracts were first filtered through clean cotton cloth and then with Whatman No. 1 filter paper. The residue was remacerated with another volume of 80% methanol and the procedure was repeated twice. Filtrates from each extraction were combined, concentrated, and dried under vacuum oven at 40°C so as to ensure complete removal of the extraction solvent. The dried crude extracts were labeled and stored at 4°C until used [20].

### 2.2. Ethical Consideration

Before start of the study, ethical clearance letter (ERC No. 1070/2017) was obtained from the Ethics Review Committee of the Research and Community Service, College of Health Science, Mekelle University.

### 2.3. Experimental Animals and Extract Administration

Female Swiss Albino mice, weighing 25–28 g and aged 8–10 weeks, were obtained from Mekelle University, College of Veterinary Sciences. Experiments were done following the internationally accepted guideline for laboratory animal care and use [21]. Mice were acclimatized for a week to the laboratory conditions before the commencement of the experiment and were provided free access of standard rodent pellet and water. They were maintained at room temperature with 12 hrs light/dark cycles until the end of the experiment. Mice were randomly distributed into three groups of ten mice each (*n* = 10). Group I received distilled water and served as a control; groups II and III received 400 and 1000 mg/kgbwt/day, respectively, of extract of *S. occidentalis* seeds that were dissolved in distilled water. Prior to dosing, the mice were fasted for 4 hr, but with a free access to water, and weighed and the extract doses were calculated based on their body weight and then were daily administered through oral route using oral gavage (stomach tube). A limit dose for toxicity testing (1000 mg/kg) [22] and a lower dose (400 mg/kg) were considered in the present investigation.

### 2.4. Measurements of Body Weight

All mice were weighed using digital balance before the beginning of the first administration and weekly until the last day of the experiment. Final body weight was recorded on the last day after 12 hrs of fasting following administration of the test extract [23].

### 2.5. Blood Collection for Biochemical Analyses

After an overnight fasting at the 29^th^ day of the experiment, all mice were anaesthetized with 70% chloroform and blood was collected immediately by cardiac puncture and transferred into labeled tubes without anticoagulant [24]. Blood samples were centrifuged for 5 min to separate serum and the separated serum was used for biochemical analyses to determine the level of liver enzyme markers: alanine aminotransferase (ALT) and aspartate aminotransferase (AST) [25].

### 2.6. Animal Dissection, Measurement of Organ Weight, Tissue Collection, and Processing

All mice were sacrificed and dissected immediately after blood collection at the 29^th^ day of the experiment. Liver of each mouse in each group was removed carefully and placed in normal saline so as to clean from unnecessary tissue remnant. Absolute weight of the liver was measured using sensitive balance. The relative organ weight was calculated from body weight and absolute organ weight of liver using the formula used elsewhere [26, 27]. The procedure described by Sangodele et al. [28] was followed with slight modifications to do tissue processing. Liver samples were immersed in 10% buffered formalin and then smaller pieces of liver tissues were taken and further fixed in 10% buffered formalin for 24 hrs to preserve and prevent the tissue pieces from degeneration. The tissue samples were washed with water and dehydrated with graded increased concentrations of alcohol. Eventually, the liver tissues were passed into two changes of absolute alcohol, two changes of xylene I and in xylene II for the purpose of clearing, and then infiltrated with two changes of paraffin wax for infiltration/impregnation. Then, tissues were embedded with paraffin wax and tissue blocks were labeled and prepared for sectioning that was sectioned with a thickness of 5 *μ*m using rotary microtome. Sections were appropriately spread on a water bath and picked using clean slides. The tissue containing slides were labeled and mounted using DPX to maximize surface adhesion, arranged in slide racks, and then placed in an oven with a temperature of 60°C for 10–15 minutes. The tissue sections were then cool dried and stained with routine hematoxylin and eosin staining technique. After examination of histological slides of all groups, photomicrographs of selected liver sections of treated and control mice were taken using digital camera installed light microscope and recorded.

### 2.7. Statistical Analysis

All the values in the test are presented as mean ± SEM. Statistical differences between the means of groups were evaluated by one-way analysis of variance (ANOVA) using the SPSS version 21 software. *p* values less than 0.05 were considered to be significant.

## 3. Results

### 3.1. Changes in Body and Organ Weight

**Figure 1:**
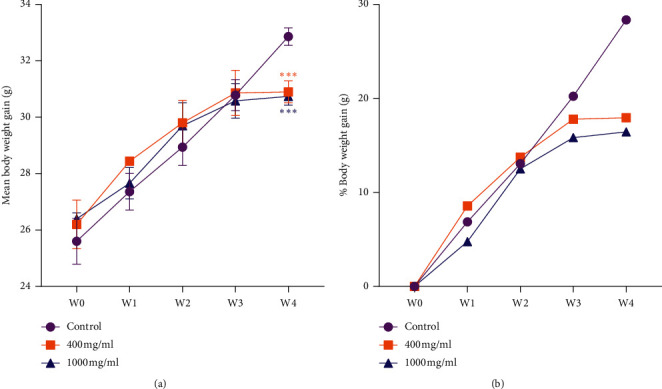
Pattern of (a) mean body weight and (b) percentage body weight gain of mice treated with methanolic extract of *Senna occidentalis* seeds as compared to control. Data are presented as mean ± SEM. Asterisks show significant difference from the control: ^*∗∗∗*^*p* < 0.001.

The mice treated with methanol extract of *S. occidentalis* seeds at doses of 400 and 1000 mg/kg did not show a statistically significant difference in their mean body weight (mbwt) gain until the third week of administration when compared to the control groups. However, in the 4^th^ week of the study period, the mbwt gain of mice treated at both doses of the extract was significantly (*p* ≤ 0.001) decreased (Figure 1(a)). As can be seen in Figure 1(b), a steady %increase (gain) in mbwt was shown for the control group, whereas for the extract treated groups the %mbwt gain started to deviate from the control group in the 3^rd^ week and the difference became obvious in the 4^th^ week. Moreover, there was a statistically significant increase in absolute and relative (to body weight) liver weight of mice treated with methanol extract of *S. occidentalis* seeds at the limit dose of 1000 mg/kg as compared to the control group (Table 1).

### 3.2. Changes on Biochemical Parameters

The serum levels of alanine aminotransferase (ALT) and aspartate aminotransferase (AST) were significantly elevated in the groups treated with the extract of *S. occidentalis* seeds at both tested doses of 400 mg/kg and 1000 mg/kg when compared to the control group (Table 2).

### 3.3. Gross Observations

During the whole period of subacute toxicity study, there was no mortality in all mice treated with the methanol extracts of the plant at the specified doses administered. Moreover, liver of treated groups did not show noticeable sign of toxicity and abnormal changes as compared to the control.

### 3.4. Histopathological Changes on Liver Tissue

**Figure 2:**
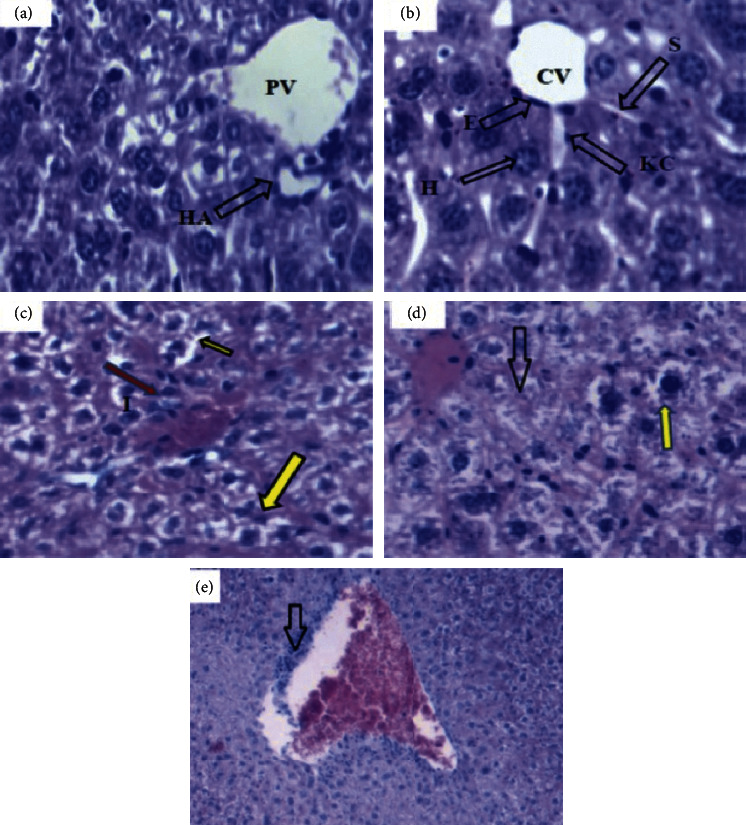
Photomicrographs of liver sections (H & E stain, X400) illustrating hepatocytes (H), central vein (CV), sinusoids (S), endothelial cells (E), kupffer cells (KC), portal vein (PV), and hepatic artery (HA). (a, b) depict liver sections of control mice showing normal architecture of liver with intact hepatic lobules and portal tract, (c) depicts liver sections of mice treated with 400 mg/kg extract of *S. occidentalis* showing moderate microvesicular steatosis of hepatocytes diffusely (yellow arrow) and mild inflammation (red), (d) depicts liver sections of mice treated with 1000 mg/kg extract of *S. occidentalis* showing marked micro- and macrovesicular steatosis (yellow arrow) and focal area necrosis (normal arrow), and (e) depicts liver sections periportal inflammation with mononuclear cell infiltration (normal arrow).

As shown in Figures 2(a)–2(e), the histopathological examination of liver sections obtained from control mice (Figures 2(a) and 2(b)) showed normal architecture with intact hepatic lobules and portal tract, whereas mice treated with methanol extract of *S. occidentalis* seeds at dose of 400 mg/kg showed moderate microvesciular steatosis of hepatocytes in a diffuse manner and mild inflammations (Figure 2(c)). Moreover, marked microvesicular and macrovesicular steatosis of hepatocytes, focal area of necrosis (Figure 2(d)), and periportal inflammation with mononuclear cells infiltrates (Figure 2(e)) were detected in mice treated with the extract of *S. occidentalis* seeds at a dose of 1000 mg/kg.

## 4. Discussion

In the present study, a significant reduction in body weight gain of mice treated with methanol extract of *S. occidentalis* seeds was exhibited in the fourth week of administration as compared to the control group (Figure 1(a)). In line with our findings, a report from a similar study showed that long-term administration of commercial feed mixed with *S. occidentalis* seeds at ration of 2% exhibited reduction of mean body weight gain in rats [7]. Likewise, studies done using chicks and rats that were fed with different rations of *S. occidentalis* showed decrease in mean body weight gain [29–31]. These evidences imply that exposure to *S. occidentalis* seeds at higher dose and/or for a prolonged time may cause harmful effects including body growth patterns. Moreover, mice treated with the limit dose (1000 mg/kg) of *S. occidentalis* seed extract showed a significant increase in both absolute and relative liver weight as compared to the control group. Equally, a similar study in rats treated with *S. occidentalis* seeds in rations of 1%, 2%, 3%, and 4% showed that relative liver weight was increased at the 4% received rats [31]. During toxicity studies, the comparison of the organ weights of treated animals with untreated animals is often complicated by the changes in body weights between groups. Yet, it was shown that regarding liver and thyroid gland weights, the best comparison of organ weights between treated animals and untreated animals is achieved using organ-to-body weight ratios [32]. In rats and mice, increases in relative (to body weight) liver weights ≤15% without further effects observed at (histo) pathology compared to concurrent controls can be considered as an adaptive nonadverse change and should not be considered as an adverse effect [33]. However, the increase in relative liver weight resulting from treatment of *S. occidentalis* seeds at 400 mg/kg and 1000 mg/kg was 54% and 126%, respectively, showing values fairly higher than 15%, and, therefore, may be considered as a manifestation of hepatotoxic effect of *S. occidentalis* seeds.

Furthermore, results of the biochemical analysis in the present study demonstrated that mice treated with *S. occidentalis* seed extract at both doses (400 and 1000 mg/kg) showed significant elevation in the serum levels of alanine aminotransferase (ALT) and aspartate aminotransferase (AST) when compared to the control group (Table 2). Since ALT and AST are primarily expressed in liver cells and are available in large amount in liver than other organs, their content is elevated during liver injury and they remain as gold standard liver injury biomarkers [34, 35]. AST is localized in the heart, brain, skeletal muscle, and liver tissue. Thus, a high level of AST not only indicates liver damage but also may be due to cardiac or muscle injury [35]. Also, the distribution of the two enzymes in liver cells differs: ALT is predominantly distributed in the cytoplasm, whereas AST is located in the cytoplasm and the mitochondria. In liver function examinations, ALT levels indicate liver cell damage and AST is a marker of liver cell necrosis [36]. Therefore, the higher fold increase of AST than ALT (Table 2) compared to control group may show that the toxicity induced by the extract is not specific to the liver and cell necrosis is implicated. Also, results of the histopathological examination of the present study indicated that daily administration of extract of *S. occidentalis* seeds at both tested doses produced signs of hepatic damage compared to the control groups, and the severity was dose-dependent. Microvesicular steatosis and mild inflammation were the main signs of injury by the 400 mg/kg group, whereas marked micro- and macrovesicular steatosis accompanied by focal area necrosis and periportal inflammation with mononuclear cell infiltration were the main signs of injury by the 1000 mg/kg extract treated group. These data are in line with previous reports. Similarly, a subacute toxicity study in rats treated with *S. occidentalis* seeds at doses of 100 mg/kg, 200 mg/kg, and 300 mg/kg presented liver section with mild vascular congestion and mild periportal infiltrates of chronic inflammatory cells [37]. Furthermore, liver section of chicks treated with 0.3% ration of *S. occidentalis* seeds showed diffuse vacuolation of hepatocytes that was more intense in the fourth week of administration [6]. Additionally, subacute treatment of rabbits with *S. occidentalis* seeds in portion of 4% showed vacuolar degeneration in their hepatocytes, with the cells presenting a foamy cytoplasm [3]. Also, despite limited reports on human intoxications, *S. occidentalis* poisoning was presented in children who ate its beans/seeds and who were reported to experience toxic liver cell necrosis with little inflammation [7]. On the other hand, toxicity studies on other parts such as stems and leaves of the study plant showed no sign of toxicity to rats [10], indicating that the seeds are the main plant part that can cause potential toxicity. Besides, the specific toxic compound responsible for the toxicity of *S. occidentalis* seeds has not been clearly characterized although some constituents like N-methylmorpholine, toxic alkaloids, anthraquinone derivatives, and toxalbumins were reported as possible candidates [1]. Therefore, further studies may be suggested that characterize the specific toxin, especially in the seeds of *S. occidentalis*. Besides, several previous studies that suggested induction of mitochondrial damage is the key mechanism by which the seeds produce toxic effects [1] and mitochondrial dysfunction has been postulated to cause nonalcoholic fatty liver disease [38]. The liver steatosis caused by *S. occidentalis* seed extract in the present study seems in line with the previous studies. Overall, the hepatotoxic potential of seeds of *S. occidentalis* appears to be obvious as was supported by experimental data of the present study revealing clear effect on body and organ weight, liver enzyme markers, and histopathological manifestations.

## 5. Conclusion

Results of the present study showed that subacute administration of extracts from *S. occidentalis* seeds caused hepatotoxicity in mice characterized by changes in liver tissue architecture and liver enzyme levels, although toxicity may not be liver-specific. These results along with supportive literature data show that high dose or long-term exposure to seeds of *S. occidentalis* may cause liver toxicity to people residing in places where this plant is prevalent as a potential crop contaminant.

## Figures and Tables

**Table 1:** Absolute and relative organ weight (g) during a 28-day subacute toxicity study on *S*. *occidentalis* seed extract compared to control group.

Groups	Absolute liver weight (g)	% increase	Relative liver weight (g)	% increase
Control	2.1 ± 0.044	0	6.4 ± 0.11	0
400 mg/kg	2.14 ± 0.089 (0.883)	4	6.94 ± 0.27 (0.204)	54
1000 mg/kg	2.36 ± 0.064 (0.036)*∗*	26	7.69 ± 0.25 (0.003)*∗*	129

Data are presented as mean ± SEM. Statistical significance is shown in parenthesis and asterisks show significant difference from the control: ^*∗*^*p* < 0.05; ^*∗∗*^*p* < 0.01.

**Table 2:** Levels of alanine aminotransferase (ALT) and aspartate aminotransferase (AST) after 28-day treatment of *Senna occidentalis* seed extract as compared to control.

Biomarkers	Control	400 mg/kg	1000 mg/kg
ALT	23.7 ± 3.85	36.2 ± 1.25 (0.045)^*∗*^	59 ± 13.1 (0.016)^*∗*^
ALT-fold increase	1.00	1.53	2.49
AST	93.4 ± 8.85	246.2 ± 50.45 (0.013)^*∗*^	295.8 ± 26.16 (0.002)^*∗*^
AST-fold increase	1.00	2.64	3.17

Data are presented as mean ± SEM. Statistical significance is shown in parenthesis and asterisks show significant difference from the control: ^*∗*^*p* < 0.05; ^*∗∗*^*p* < 0.01.

## Data Availability

All the data used and analyzed during the present study will be available from the corresponding author if deemed necessary.

## Abbreviations

ALT: Alanine aminotransferase
ANOVA: One-way analysis of variance
AST: Aspartate aminotransferase
bwt: Body weight
DPX: Dibutyl phthalate in xylene
H & E: Hematoxylin and eosin
kg: Kilogram
mg: Milligram
SEM: Mean standard err.
